# P-451. Adherence to Bone Mineral Density Screening Recommendations in Older Adults with HIV

**DOI:** 10.1093/ofid/ofae631.651

**Published:** 2025-01-29

**Authors:** Madison Martz, Nazar Akhverdyan, Melissa P Wilson, Jacob Walker, Sarah Gorvetzian, Lakshmi Chauhan, Kristine M Erlandson

**Affiliations:** University of Colorado School of Medicine, Aurora, Colorado; University of Colorado School of Medicine, Aurora, Colorado; University of Colorado, Aurora, Colorado; University of Colorado, Aurora, Colorado; University of Colorado, Aurora, Colorado; University of Colorado, Aurora, Colorado; University of Colorado Anschutz Medical Campus, Aurora, CO

## Abstract

**Background:**

HIV guidelines recommend bone mineral density (BMD) screening by dual-energy x-ray absorptiometry (DXA) for all postmenopausal women and all men ≥50 years. Uptake of these recommendations has been low. This study describes DXA screening practices among people with HIV (PWH) at highest risk for low BMD.

**Methods:**

We conducted a retrospective chart review among PWH aged ≥65 or older who were seen for HIV care at the University of Colorado within the last year. First available and most recent DXA scans were collected; FRAX score was calculated to estimate 10-year risk of major osteoporotic and hip fractures. Demographics and clinical factors were also collected.

**Results:**

We reviewed records of 300 PWH age 65 and older, including 243 (81%) men and 57 (19%) women. The mean age was 69.8 (SD 4.4) years, 62% were non-Hispanic white, 45% had a normal body mass index (18.5-24.9 kg/m^2^), and 60% reported current/prior tobacco use. 48% had a DXA scan ordered, including 53% of those with previous falls and 54% with prior fracture. Mean age at first DXA scan was 64.9 (SD 6.0) for males and 64.5 (SD 6.4) for women. Of those screened, 13% of women and 27% of men had normal BMD (T-score > -1.0); 45% of women and 53% of men had osteopenia (T-score < -1 to -2.5) and 42% of women and 20% of men had osteoporosis (T-score < -2.5). By FRAX, 10-year probability of major osteoporotic fracture was 9.8% [IQR: 5.7, 13.6] for women and 6.8% [4.7, 11.0] for men. The 10-year probability of hip fracture was 2.2% [0.8, 4.1] for women and 1.4% [0.6, 2.8] for men.

**Conclusion:**

Older PWH are markedly under-screened for low BMD. There was low adherence to screening per current HIV guidelines, even among those with the highest risk. Osteopenia and osteoporosis were common, though FRAX scores suggested only moderate risk. Improved screening for reduced BMD and fracture risk in PWH is urgently needed.

**Disclosures:**

**Kristine M. Erlandson, MD MS**, Gilead: Advisor/Consultant|Gilead: Grant/Research Support|ViiV: Advisor/Consultant

